# Interpretation of the Top-of-Atmosphere Energy Flux for Future Arctic Warming

**DOI:** 10.1038/s41598-019-49218-6

**Published:** 2019-09-10

**Authors:** Jiwon Hwang, Yong-Sang Choi, Changhyun Yoo, Yuan Wang, Hui Su, Jonathan H. Jiang

**Affiliations:** 1 Department of Climate and Energy Systems Engineering, Ewha Womans University, Seoul, Republic of Korea; 2 Jet Propulsion Laboratory, California Institute of Technology, Pasadena, CA USA; 3 Division of Geological and Planetary Sciences, California Institute of Technology, Pasadena, CA USA

**Keywords:** Atmospheric dynamics, Atmospheric dynamics, Atmospheric dynamics, Atmospheric dynamics, Attribution

## Abstract

With the trend of amplified warming in the Arctic, we examine the observed and modeled top-of-atmosphere (TOA) radiative responses to surface air-temperature changes over the Arctic by using TOA energy fluxes from NASA’s CERES observations and those from twelve climate models in CMIP5. Considerable inter-model spreads in the radiative responses suggest that future Arctic warming may be determined by the compensation between the radiative imbalance and poleward energy transport (mainly via transient eddy activities). The poleward energy transport tends to prevent excessive Arctic warming: the transient eddy activities are weakened because of the reduced meridional temperature gradient under polar amplification. However, the models that predict rapid Arctic warming do not realistically simulate the compensation effect. This role of energy compensation in future Arctic warming is found only when the inter-model differences in cloud radiative effects are considered. Thus, the dynamical response can act as a buffer to prevent excessive Arctic warming against the radiative response of 0.11 W m^−2^ K^−1^ as measured from satellites, which helps the Arctic climate system retain an Arctic climate sensitivity of 4.61 K. Therefore, if quantitative analyses of the observations identify contribution of atmospheric dynamics and cloud effects to radiative imbalance, the satellite-measured radiative response will be a crucial indicator of future Arctic warming.

## Introduction

The Arctic region (60°N–90°N) has warmed twice as fast as the rest of the globe^1–4^. This so-called Arctic amplification is known to owe its existence to the high sensitivity of the Arctic climate to climate forcing^5^. This high sensitivity can be attributed to positive radiative-feedback processes that operate over the Arctic region. The ice–albedo feedback, for example, occurs because of the reduction in sea ice, which enhances shortwave radiation absorption by opening up the dark ocean. This feedback process is often considered the leading cause of the high climate sensitivity of the Arctic^6–10^. For longwave radiation, the feedback processes can operate in association with the vertical structure of warming and changes in water vapor. That is, regionally enhanced lapse-rate and water vapor feedbacks reduce the outgoing longwave radiation, resulting in Arctic amplification^11–13^. Other studies suggested that biogeophysical feedbacks can further enhance Arctic warming via phytoplankton or vegetation changes^14^. Despite recent advances in our understanding of the Arctic climate in relation to these radiative feedbacks, the prediction of future Arctic warming still remains highly uncertain in contemporary climate models.

To validate the degree of modeled future global warming in response to rising greenhouse gases, i.e., climate sensitivity (CS), recent studies have attempted to use satellite observations of the top-of-atmosphere (TOA) radiative fluxes. For the globe (where no consideration of atmospheric energy transport across the boundary is needed), the CS can be estimated from the linear regression between changes in the global-mean energy budget and global-mean surface-temperature changes based on the Gregory method^15,16^. However, some studies noted that a deeper understanding of the regional climate physics linking the response of TOA radiative fluxes to surface warming is required prior to quantifying the global CS because the global CS is substantially influenced by regional climate-feedback processes and different periods^17,18^.

When a regional domain, such as the Arctic, is chosen, the atmospheric and oceanic energy influx/outflux at the boundary of the domain should be considered to understand the regional climate physics. Changes in the regional surface temperature are naturally closely related to changes in the (vertical) TOA radiative flux and (horizontal) dynamical energy-flux^19^. The observed monthly change in TOA radiative fluxes in response to surface air-temperature change (hereafter, radiative response) over the Arctic could be decomposed into individual radiative-feedback components, such as the surface albedo, water vapor, lapse rate, and clouds^20^. However, these radiative-feedback components alone cannot thoroughly explain the present-day radiative response because residual-feedback parameters, which are calculated by subtracting the sum of individual feedback parameters from the radiative response, represent relatively large values^20^. Therefore, some other processes, especially the role of horizontally transported dynamical energy-flux, should be considered to estimate future Arctic temperature changes^21,22^.

Coupled climate-model experiments revealed that regional radiative-feedback processes contribute more to future Arctic temperature changes than atmospheric-energy transport^23,24^. Seemingly opposed to these studies, recent studies suggested that atmospheric-energy transport from lower latitudes to the Arctic is rather critical to future Arctic warming^25,26^. In addition, both observations^27^ and model simulations^28^ demonstrated that Arctic amplification will be alleviated when the sea-ice concentration reaches a critical level of 10–20%, which is attributable to the possibility of the increasing role of regional heat-flux changes when the contribution from albedo feedback to Arctic amplification decreases^27,28^.

These previous studies imply that the Arctic radiative response is linked to changes in atmospheric-energy transport into the Arctic, which is controlled by dynamical processes such as large-scale planetary waves, mesoscale cyclones, and the zonal-mean circulation^29^. Hence, dynamically driven energy flux (hereafter, dynamical response) can accelerate or decelerate Arctic warming^30,31^. In summary, the degree of future Arctic warming should be controlled by the balance between local radiative-feedback processes and horizontally transported dynamical energy-flux changes.

Given this consideration, this study attempts to relate Arctic climate sensitivity (ACS) to satellite observations of TOA radiative fluxes, serving as an observational estimate of ACS. We can also explain the role of dynamical energy flux in ACS. To these ends, this study uses Clouds and Earth’s Radiation Energy System (CERES)-retrieved TOA radiative fluxes and the corresponding outputs of twelve climate models participating in the Fifth Phase of the Coupled Model Intercomparison Project (CMIP5).

## Results

### Standard energy-balance framework for the Arctic climate system

We extend the linearization of the global TOA energy-budget analysis to the Arctic by adding the perturbation of horizontally transported dynamical energy-flux into the domain^15,16,23,32^. The energy imbalance of the Arctic climate system is the residue of the external radiative forcing, surface air-temperature response, and change in horizontally transported dynamical energy-flux:

1 $${\rm{R}}={\rm{F}}-\frac{{\rm{T}}}{{\rm{ACS}}}+{\rm{DEF}},$$

where R is the perturbation of the downward net radiation at the TOA (if R is positive, the system gains energy). The climate forcings, such as changes in the atmospheric concentration of CO_2_ and randomly varying clouds, are represented by F. The ACS is the equilibrium response of the Arctic surface air temperature to a doubling in the CO_2_ concentration and has the unit K (W m^−2^)^−1^. Combined with T, the ACS term (i.e., –T/ACS) represents the radiative response that is controlled by ACS. T denotes the emission temperature of the Arctic climate, indicating the accumulation of heat in the climate system. Here, we use the 2-m temperature anomalies averaged over the Arctic, which is the indicator of the climate state^33^. Lastly, for a regional domain such as the Arctic, we must include the energy fluxes through the lateral boundaries with the atmosphere and ocean^17,20^. The change in such dynamical energy-flux is included as “DEF”. Each term in Eq. (1) has the unit W m^−2^.

To examine the radiative response to surface air-temperature changes, we divide Eq. (1) with T and take the time derivative d as follows:

2 $${\rm{RRC}}=\frac{{\rm{dF}}}{{\rm{dT}}}-\frac{1}{{\rm{ACS}}}+\mathrm{DRC},$$

where ACS is assumed to be independent of T. The radiative response coefficient (RRC) is defined as the TOA radiative response to changes in surface air temperature, while the dynamical response coefficient (DRC) is defined as the response of the DEF to changes in surface air temperature:

2a $${\rm{RRC}}\equiv \frac{{\rm{dR}}}{{\rm{dT}}}$$

and

2b $${\rm{DRC}}\equiv \frac{{\rm{dDEF}}}{{\rm{dT}}},$$

where the derivative d means the time-dependent perturbation. In practice, the RRC is estimated from the regression slope of the monthly R on the monthly T^34–38^. However, calculating the DRC with the given variables (e.g., TOA radiative fluxes and surface air temperature) is difficult because changes in the TOA radiative flux are non-linearly associated with horizontally transported dynamical energy-flux, such as dry static energy flux or latent heat flux. Instead, the DRC is derived through Eq. (2) from the pre-calculated values of the RRC and ACS and are assumed to partially originate from the energy transport of transient eddy activities in the Arctic region^39^.

The rate of change in the radiative forcing (i.e., dF/dT in Eq. (2)) can be ignored because the change in external CO_2_ forcing during the short time span in this study can be assumed to be relatively small^37^. Other internal natural forcings, such as randomly varying clouds, are independent of surface air-temperature change. Therefore, the ACS is the reciprocal function of the difference between the RRC and DRC:

3 $$\frac{1}{{\rm{ACS}}}=-\,{\rm{RRC}}+{\rm{DRC}}$$

In this study, the RRC is estimated based on both satellite observations and CMIP5-model outputs, and the ACS is calculated from CMIP5-model outputs. The detailed calculation method of the RRC and ACS will be explained in the Methods section.

### Physical concept of the RRC and the RRC-DRC relationship

**Figure 1:**
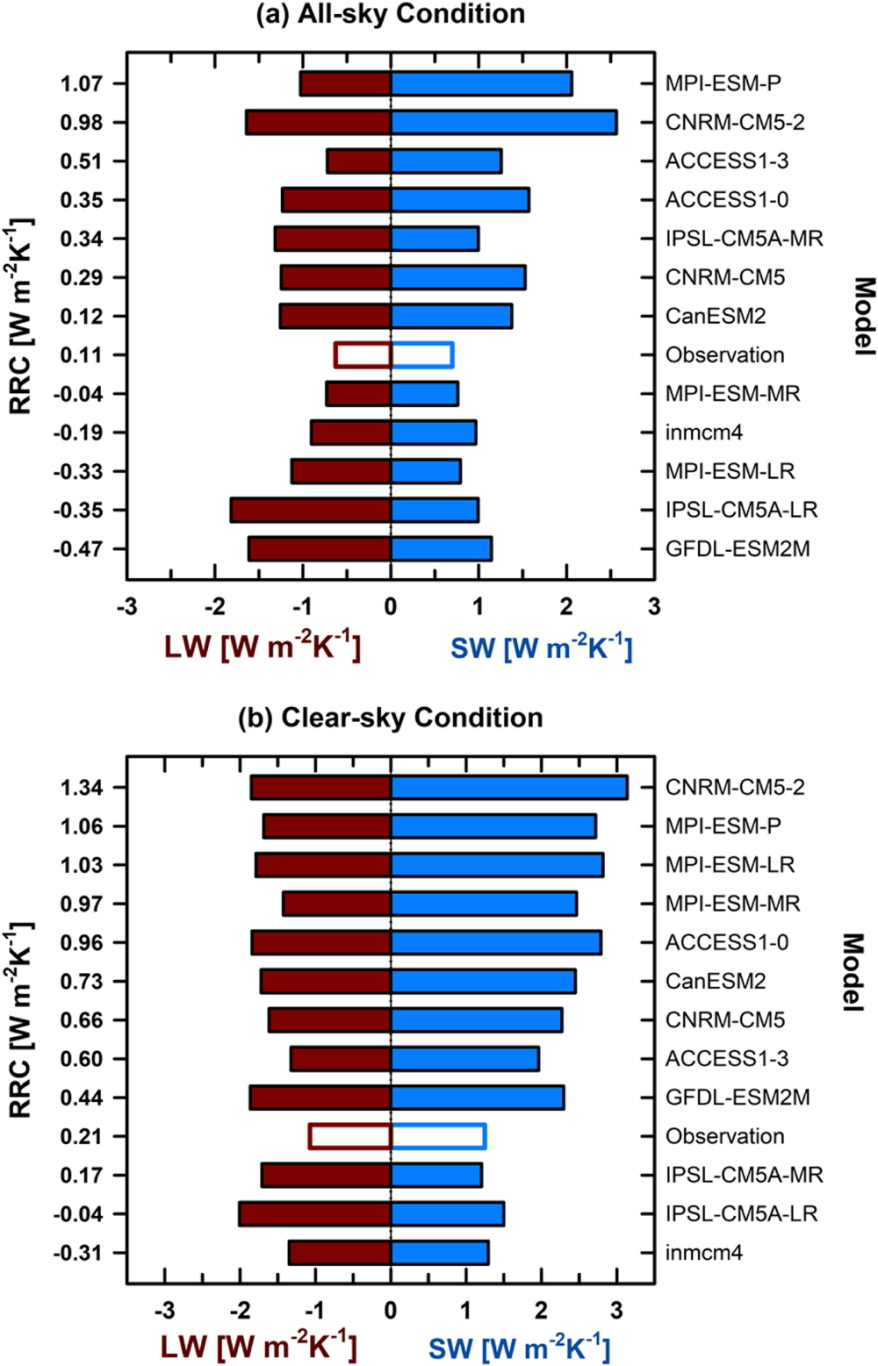
Inter-model spreads in the shortwave (SW) and longwave (LW) radiative-response coefficients (RRC) given an increase in the net RRC under (**a**) all-sky and (**b**) clear-sky conditions for the 12 CMIP5 models.

To understand the physical meaning of the RRC, the contributions from longwave (LW) and shortwave (SW) radiation to the RRC are compared for each model, and cloud effects are examined by comparing the all-sky and clear-sky conditions. Figure 1 represents the observed and inter-model spreads of the RRC. The RRC ranges from negative to positive values, with positive values indicating anomalous increases in the incoming (or anomalous decreases in the outgoing) TOA net radiative flux to surface air warming and negative values indicating anomalous increases in the outgoing TOA net radiative flux to surface air warming. A negative LW response includes the Planck feedback. The observational RRC value (0.11 W m^−2^K^−1^) falls within the model range (−0.47 to 1.07 W m^−2^ K^−1^), and the models in the middle ranks of the RRC (CanESM2 and MPI-ESM-MR) are not statistically different with the observational RRC. Therefore, these models should approximately reflect the radiative response of the real Arctic climate system. In addition, the observational RRC value that was estimated in this study is somewhat consistent with the total radiative response according to recent research (0.55 ± 0.54 to 1.55 ± 0.62 W m^−2^K^−1^)^38^ by replacing the TOA radiative fluxes from CERES with the Community Atmosphere Model version 5 or three different reanalysis products. Under all-sky conditions, the models with larger RRC values are more strongly influenced by the SW response. Meanwhile, the models with smaller RRC values are more strongly influenced by the LW response (Fig. 1a).

In contrast, the LW and SW radiative responses in the models have small variances under clear-sky conditions (Fig. 1b). Except for the SW RRC in the models with lower net RRC, the variances of SW and LW RRCs in the all-sky condition tend to be larger than that in the clear-sky condition (not shown). This different radiative response under clear- and all-sky conditions demonstrates that clouds significantly influence the modeled RRC in the Arctic region. This inter-model diversity of the radiative response under all-sky conditions is probably caused by considerable model differences in the interaction between clouds and the sea-ice albedo because various changes in clouds affect the sea-ice surface-energy budget in models differently^40^.

**Figure 2:**
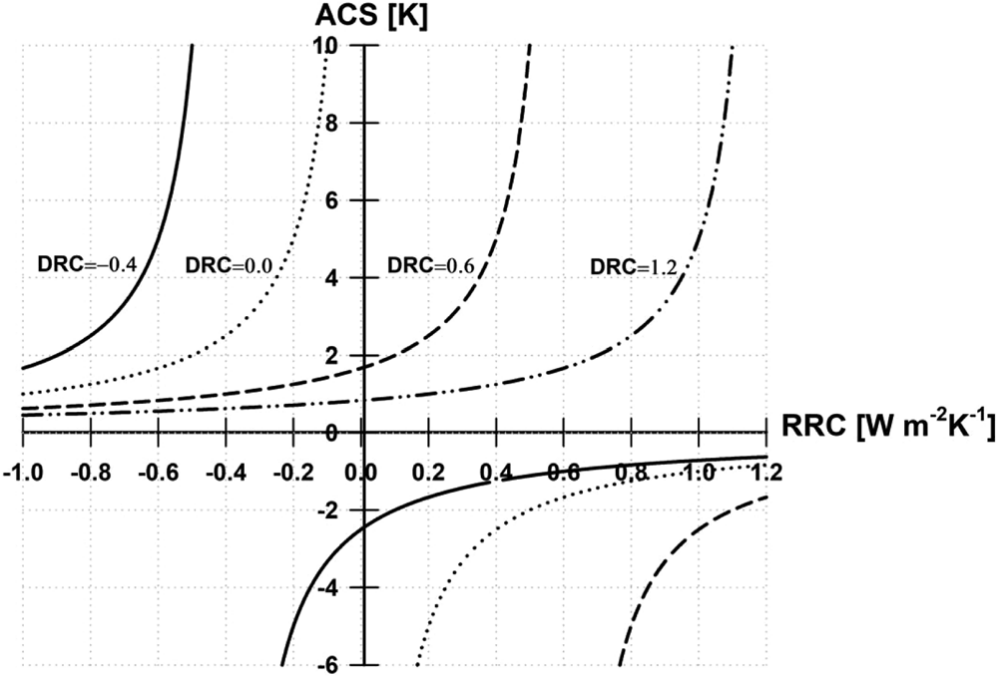
Diagram of the Arctic climate sensitivity (ACS) as a function of the Arctic radiative-response coefficient (RRC) for four values of the dynamical response coefficient (DRC) based on Eq. (3).

Figure 2 illustrates a schematic picture of the relationship between the RRC and ACS for different DRCs. The graph that links ACS to the RRC shows the same shape as in a previous study that demonstrated the relationship between “global” CS to feedback factors^41^. However, for the Arctic, DRC plays a role in controls the rate of ACS changes to RRC. The RRC ranges from negative to positive values, and the ACS increases with the RRC following Eq. (3). For relatively small DRC values (dotted line), the ACS rises sharply with an increasing RRC. For large DRC values (dashed double-dotted line), the ACS hardly changes with an altered RRC within the same range of RRC values. The ACS, meanwhile, exhibits extreme values if the RRC is sufficiently large. When the RRC becomes zero, the ACS retains a value greater than zero, which means that horizontally transported dynamical energy-flux maintains positive ACS without a radiative response. If the RRC is greater than or equal to the DRC, the ACS shows unphysical conditions with negative ACS values (solid line in the third and fourth quadrants in Fig. 2); hence, the DRC should always be greater than the RRC in the Arctic climate system. Thus, the change in poleward energy transport from atmospheric or oceanic currents is greater than the radiative response at the TOA as the surface air temperature rises because the DRC is affected by both local climate feedbacks and remote effects, such as Arctic–mid-latitude teleconnections^42^. Under global warming, changes in sea-surface temperatures and radiative-feedback processes at low latitudes can enhance poleward energy transport through atmospheric circulation to higher latitudes, which may amplify Arctic warming^43^.

One must be cautious that the relationship between the DRC and RRC may not be independent but complementary and interdependent. An energy imbalance could cause energy influx to the Arctic, which tends to reduce this imbalance. Consequently, the RRC could be non-linearly related to the DRC, and the difference between the DRC and RRC would determine the ACS. In the next section, we investigate this relationship between the RRC and ACS from CMIP5-model simulations.

### Determination of the Arctic climate sensitivity

**Figure 3:**
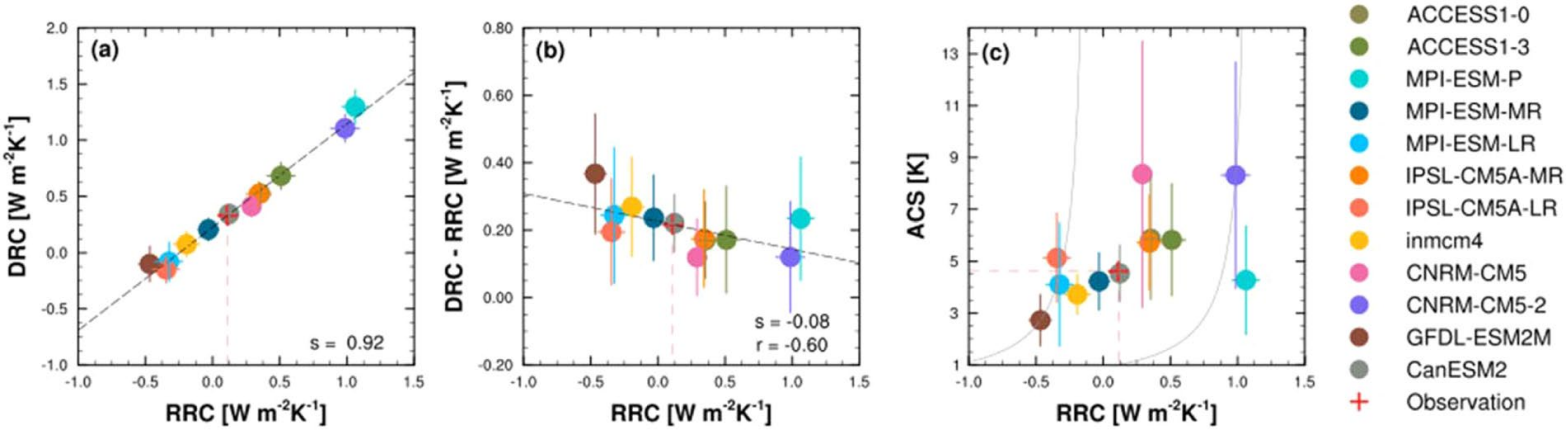
Arctic radiative-response coefficient (RRC) against the (**a**) dynamical-response coefficient (DRC); (**b**) DRC–RRC, which is identical to the inverse of the Arctic climate sensitivity (ACS); and (**c**) ACS of 12 CMIP5 models. The *black dashed line* indicates the regression line between the RRC and each Y-axis value. The *red dashed line* represents the estimated RRC from satellite observations, and the *red cross symbol* indicates the corresponding values on the regression line. The *gray solid lines* represent the theoretical relationship between the RRC and ACS for GFDL-ESM2M and CNRM-CM5-2. The *error bars* indicate the standard error of the regression coefficients. These relationships could not be found under clear-sky conditions.

Each model’s RRC is also estimated from the TOA radiative response to surface air-temperature changes in transient historical experiments, and each model’s ACS can be calculated from the temperature response to a doubling in CO_2_ forcing based on abrupt 4xCO_2_ and pi-Control experiments. Each model’s DRC can be derived from the relationship between the ACS and RRC. An inter-model comparison of the RRC and DRC identified that model with larger radiative responses tend to show larger dynamical responses (Fig. 3a). MPI-ESM-P and CNRM-CM5-2 exhibit large RRC and DRC values, while IPSL-CM5A-LR, GFDL-ESM2M, and MPI-ESM-LR indicate relatively small RRC and DRC values. In addition, if the observational RRC value (0.11 W m^−2^ K^−1^) is plotted on the regression line, the DRC is 0.33 W m^−2^ K^−1^ (following the red dashed line in Fig. 3a). Consequently, the current climate models demonstrate that the DRC is comparable to the RRC because poleward atmospheric-energy transport mediates the interaction between the surface-albedo and lapse-rate feedbacks^44^.

More importantly, the DRC minus the RRC (DRC–RRC) appears to be negatively related to the RRC (r RC-0.6) in Fig. 3b. A large RRC requires a small DRC–RRC, and the DRC–RRC values range from 0.0 to 0.4 W m^−2^ K^−1^. This negative relationship is apparently shown for RRCs that are estimated under all-sky conditions but not for RRCs that are estimated under clear-sky conditions (figure not shown). Hence, the relationship between the RRC and DRC–RRC might be associated with model differences in terms of cloud radiative effects.

The ACS of each model is then compared to DRC–RRC (Fig. 3b,c). The models with relatively small DRC–RRC values tend to have high ACS, while the models with large DRC–RRC values tend to have low ACS. In addition, the relationships among the RRC, DRC, and ACS can be applied to the current Arctic climate system. The ACS is proportional to the RRC (following the gray line in Fig. 3c), so the ACS can be estimated from the RRC based on satellite observations. In Fig. 3c, the observational RRC is marked as a red dashed line for the value of 0.11 W m^−2^ K^−1^. If the DRC is large enough to compensate the RRC (corresponding to a large DRC–RRC value in Fig. 3b), the Arctic climate warms slowly (low ACS); if the DRC does not compensate the RRC (corresponding to a small DRC–RRC value in Fig. 3b), the Arctic climate warms rapidly (high ACS). Although the actual DRC is not assured in this study, this value may be inferred from an inter-model comparison to be approximately 0.33 W m^−2^ K^−1^ (Fig. 3a). Given the RRC and DRC values, the current Arctic climate sensitivity would be 4.6 K.

**Figure 4:**
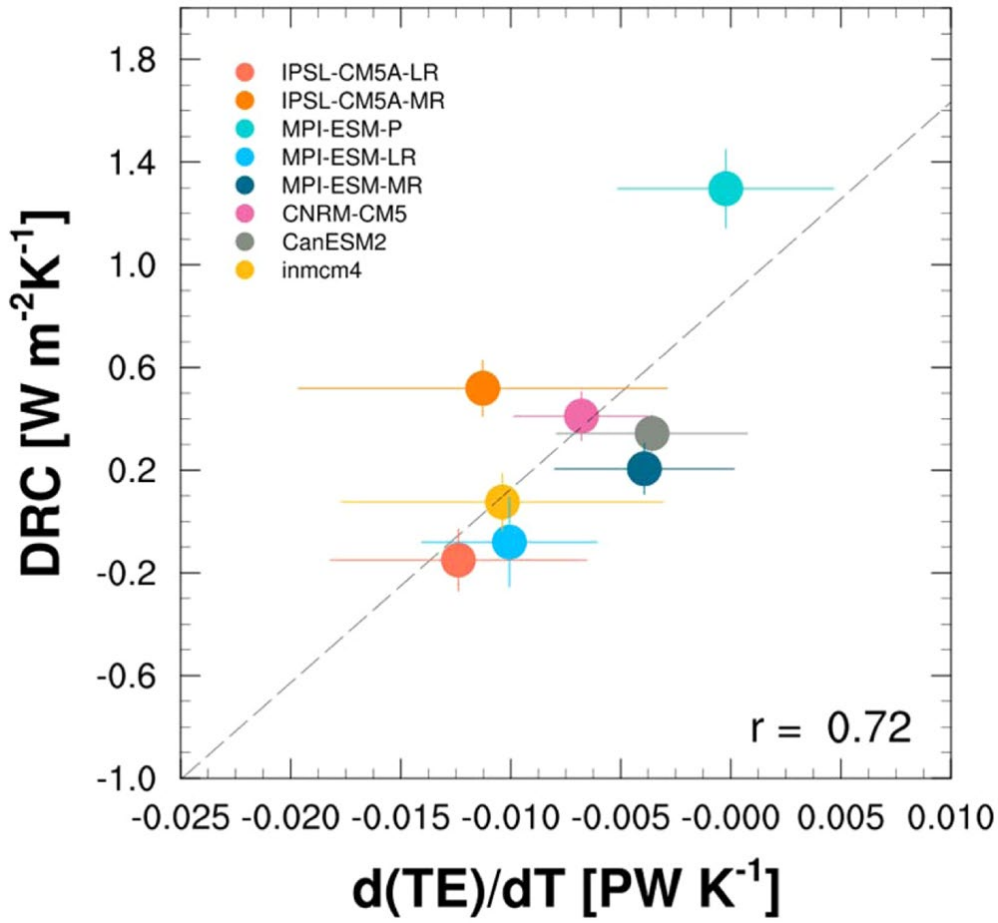
Dynamical-response coefficient (DRC) in units of W m^−2^K^−1^ against the perturbation of poleward energy transport by transient eddies (TE) to the surface-temperature change in units of PW K^−1^ for 8 CMIP5 models. By using the daily northward-wind velocity, air temperature, geopotential, and specific humidity, the poleward energy transport by transient eddies was derived from deviations from the time averages at 60°N and then vertically and zonally integrated^46^. Here, d means the derivative of the monthly anomalous perturbation. If MPI-ESM-P is excluded, the correlation coefficient would be 0.58. The *error bars* indicate the standard error of the regression coefficients.

The derived DRC from the energy-balance model includes the energy transport from both atmospheric and oceanic currents. At this moment, we investigate whether the DRC is actually related to the atmospheric current. Atmospheric energy transport originates from atmospheric waves, such as large-scale planetary waves and mesoscale cyclones^39,45^, so this study analyzes changes in poleward energy transport by transient eddies in response to Arctic warming (dTE/dT) with eight CMIP5 models (Fig. 4) (the remaining four models are excluded as they do not contain daily historical run outputs). Transient eddies (units of PW) are vertically and zonally integrated as ${\int }_{0}^{2\pi }[\frac{1}{g}{\int }_{1000}^{10}({C}_{p}v^{\prime} t^{\prime} +Lv^{\prime} q^{\prime} +gv^{\prime} z^{\prime} )dp]dx$^46^. All the negative dTE/dT values in the horizontal axis imply that the poleward energy transport from transient eddies decreased in response to Arctic warming. As the Arctic warms, poleward transient eddies weaken in response to the reduced equator-to-pole temperature gradient^47^. The positive slope of the regression line in Fig. 4 indicates that the greater dTE/dT values (i.e., smaller declines in transient eddies from Arctic warming) are responsible for the greater DRC values. In addition, poleward energy transport might be accomplished by active eddies, which control the TOA radiative balance^48^. Therefore, we conclude that transient eddies resiliently modulate the atmospheric energy transport to the Arctic, and the DRC could act as a buffer against the radiative response.

## Conclusions and Discussion

The ACS was determined from the balance between the radiative and dynamical responses based on the standard energy-balance framework for the Arctic climate system. Accordingly, we investigated the relationship among the RRC, DRC, and ACS in twelve CMIP5 general-circulation models. First, the RRC values, which were determined from regional radiative-feedback processes, varied between models. When comparing the RRCs between all-sky and clear-sky conditions, the cloud radiative effect explained the inter-model diversity in the RRCs. In particular, the SW radiative response under all-sky conditions strongly differed among the models because of the different parameterizations of the ice albedo–cloud–radiation interactions^49^, implying that changes in SW radiation considerably affect the RRC. Next, larger DRCs, which were induced by dynamically transported atmospheric-energy flux, were linked to larger RRCs. Additionally, transient poleward eddy is known to modulate the DRC. If transient-eddy activities largely decrease, the response of horizontally transported dynamical energy-flux to Arctic warming would be small. Therefore, we can conclude that changes in the incoming TOA radiative flux interact with changes in poleward transport of dynamical energy flux via a reduction in transient eddy activity.

However, we cannot quantify the contribution from atmospheric dynamics to Arctic warming, which is directly related to the DRC, because the poleward dry static and latent energy fluxes, which are driven by eddies, compensate one another^26^, and the contributions from eddy fluxes are different in terms of energy components^50^. Moreover, transient and stationary eddies counteract each other^51^. We only analyzed the average transient poleward eddies over the entire Arctic region and the annual mean, so the quantitative contribution from transient poleward eddies to the DRC is still unclear. Furthermore, the effect of oceanic heat flux on the DRC was not considered in this study because the contribution from oceanic transport to the total transport is relatively smaller than the atmospheric energy transport at 60°N^52^. However, recent studies suggested that the perturbed ocean heat flux at lower latitudes can affect Arctic warming, with increased poleward energy transport^43,53^. Hence, a careful analysis of changes in the transient and stationary eddies in different seasons and regions and the role of oceanic heat transport should be further investigated to determine the source of the DRC. In addition, quantifying the DRC values depends on linear assumptions following the standard energy-balance framework; therefore, one must be cautious that an indirectly calculated DRC might have uncertainty because of nonlinearity.

The inter-model spreads in the RRC and ACS demonstrated that the difference between the DRC and RRC determined the ACS. Models with high climate sensitivity were more likely to have small differences between the DRC and RRC, whereas models with low sensitivity tended to have large differences between the DRC and RRC. Thus, the DRC mediated the climate sensitivity against the RRC in the Arctic. This finding was also confirmed in previous studies, in which atmospheric-energy transport was negatively associated with changes in polar-climate sensitivity^12,30^. In addition, Arctic warming is known to slow down in regions with strong poleward atmospheric-energy transport as sea ice decreases^27^.

Therefore, if the DRC is accurately quantified, the TOA radiative response that is observed by satellites will be an accurate indicator of future Arctic warming. However, RRCs that are derived from a state-of-the-art climate model cannot be directly compared to RRCs that are estimated from satellite sensors. The former are based on historical periods (1981–2005), while the latter are from satellite-operation periods (2000–2016); additionally, historical simulations of the CMIP5 model cannot cover the early 2010s, when Arctic sea ice was observed to have reached its minimum extent. Therefore, the relationship between the ACS and RRC in each climate model may involve uncertainty. However, as CMIP6 currently extends the period up to 2014, these limitations of this study may be overcome in the future work. Nevertheless, this study revealed the linkage between Arctic climate sensitivity and satellite observations of the TOA radiative response, which is controlled by compensation effects from atmospheric dynamical energy transport.

## Methods

### Description of the satellite data and climate-model simulations

To investigate the radiative response from satellite observations, monthly TOA radiative fluxes, including the incident solar radiation (ISR), outgoing shortwave radiation (OSR), and outgoing longwave radiation (OLR), were obtained from the CERES Energy Balanced and Filled (EBAF) Edition 4 products^54^ for the 17-year period of March 2000–December 2016. Additionally, monthly surface air-temperature data at 2-m height (SAT) were collected from the ERA-Interim reanalysis dataset produced by the European Centre for Medium-Range Weather Forecasts (ECMWF) for the same period^55^.

Then, we selected fifteen models from the CMIP5 multi-model data archive (http://cmip-pcmdi.llnl.gov/cmip5/index.html), including historical, abrupt CO_2_ quadrupling (4xCO_2_), and pre-industrial control (pi-Control) experiments. The monthly TOA ISR, OSR, OLR, and SAT from the historical-simulation outputs for the 25-year period of 1981–2005 were used to analyze the inter-model spread of the radiative response, and the annually averaged variables from the 4xCO_2_ and pi-Control outputs were used to calculate the modeled ACSs. To calculate the dynamical response, daily averages of the vertical profiles of the zonal wind (u), temperature (t), geopotential height (z), and specific humidity (q) from the historical simulations were also analyzed.

### Calculation of the RRC and ACS

**Figure 5:**
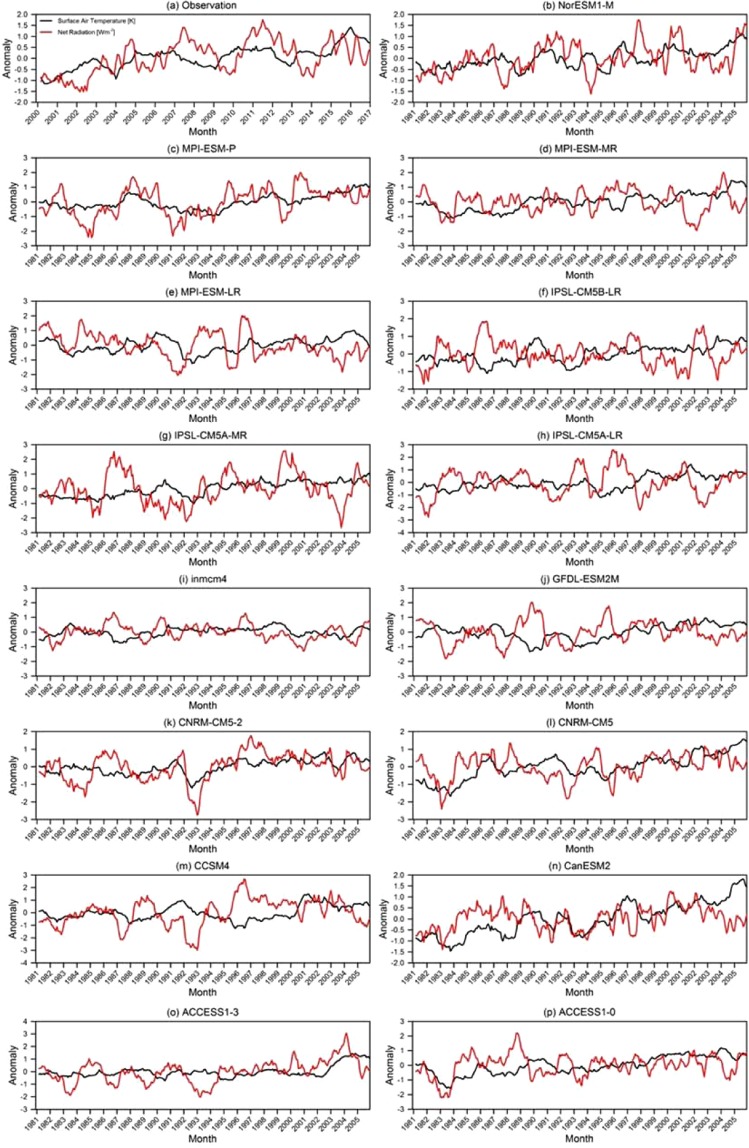
Twelve-month moving-averaged anomalous time series of the downward net radiation (red) and surface air temperature (black) in the Arctic from (**a**) CERES satellite observations for the 17-year period of March 2000–December 2016 and (**b–p**) historical simulations of 15 CMIP5 models for the 21-year period of 1981–2005.

Based on Eq. (2a), the RRC was estimated from the regression slope of R on T by using satellite observations (Fig. 5a) and the CMIP5-model outputs (Fig. 5b–p) under the assumption that any TOA radiation changes (red) were largely a response to the temperature (black) in the Arctic region. We applied monthly time-dependent perturbations to estimate a reliable RRC over the Arctic region. If time-dependent perturbations with short time scales (a few hours to days) were applied, some of R’s response to T may not have excluded long-term feedback processes, such as the ice-albedo feedback. Similarly, time-dependent perturbations with long time scales (over a month) may have included an equilibration effect of R^34,35^. Next, we took 12-month moving averages over monthly anomalous time series to exclude any non-feedback factors (e.g., strong seasonality and autonomous cloud variations) while retaining any radiative-feedback processes with time spans longer than a month, such as the ice-albedo feedback process^56^. If the 12-month moving average was not taken, non-feedback factors would have made the radiative response unstable^20^.

**Figure 6:**
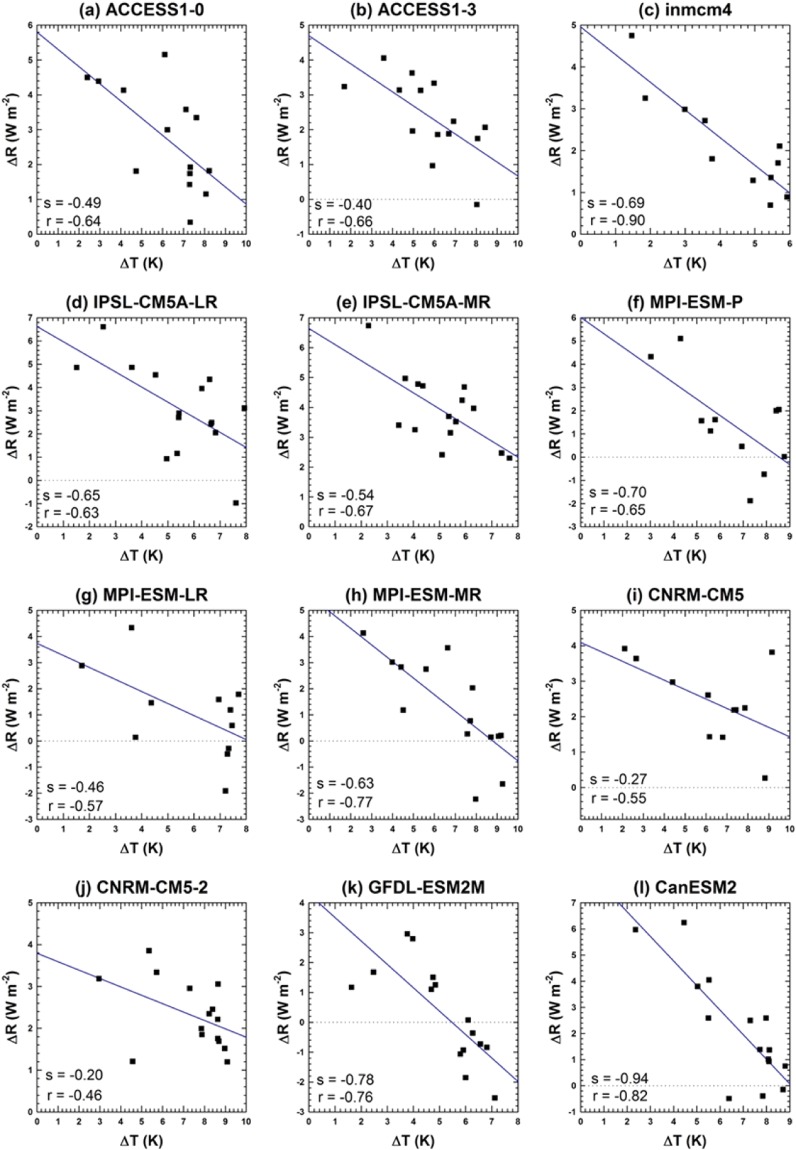
Relationships between the change in net TOA radiative flux (∆R) and surface-air-temperature change (∆T) after an abrupt quadrupling in CO_2_ in 12 CMIP5 models. The data points are Arctic annual averages. The blue lines represent simple linear-regression fits with slopes (s) and correlation coefficients (r). Here, ∆ means the difference between the abrupt 4xCO_2_ and pi-Control experiments.

The ACS was estimated from a simple linear regression of the Arctic-annual-mean TOA radiative flux (R) against the SAT after CO_2_ quadrupling^57^. R was calculated from the ISR, OSR, OLR, and poleward energy transport (PET) following the equation R = ISR-OSR-OLR + PET. Figure 6 shows a simple linear regression of ∆R against ∆T for the first few years (10 to 15 years) after CO_2_ quadrupling. ∆ means the difference between the abrupt 4xCO_2_ and pi-Control experiments. To examine the reliable temperature response to CO_2_ forcing, we did not analyze the entire experimental period because any temperature changes were largely a response to TOA radiative flux over the first few years. Afterwards, these changes non-linearly fluctuated to approach the equilibrium state, and the correlation coefficient between the two variables gradually decreased. The intercept of the regression line at ∆T = 0 measures the radiative forcing, F, while the regression slope, λ, implies the climate-feedback parameter. Accordingly, the ACS was derived as F/2λ because the CO_2_ concentration was quadrupled (not doubled)^57^.
